# High-density SNP-based genetic maps for the parents of an outcrossed and a selfed tetraploid garden rose cross, inferred from admixed progeny using the 68k rose SNP array

**DOI:** 10.1038/hortres.2016.52

**Published:** 2016-10-26

**Authors:** Mirjana Vukosavljev, Paul Arens, Roeland E Voorrips, Wendy PC van ‘t Westende, GD Esselink, Peter M Bourke, Peter Cox, W Eric van de Weg, Richard GF Visser, Chris Maliepaard, Marinus JM Smulders

**Affiliations:** 1 Wageningen UR Plant Breeding, Wageningen University & Research, NL-6700 AJ Wageningen, The Netherlands; 2 Roath BV, Eindhoven, The Netherlands

## Abstract

Dense genetic maps create a base for QTL analysis of important traits and future implementation of marker-assisted breeding. In tetraploid rose, the existing linkage maps include <300 markers to cover 28 linkage groups (4 homologous sets of 7 chromosomes). Here we used the 68k WagRhSNP Axiom single-nucleotide polymorphism (SNP) array for rose, in combination with SNP dosage calling at the tetraploid level, to genotype offspring from the garden rose cultivar ‘Red New Dawn’. The offspring proved to be not from a single bi-parental cross. In rose breeding, crosses with unintended parents occur regularly. We developed a strategy to separate progeny into putative populations, even while one of the parents was unknown, using principle component analysis on pairwise genetic distances based on sets of selected SNP markers that were homozygous, and therefore uninformative for one parent. One of the inferred populations was consistent with self-fertilization of ‘Red New Dawn’. Subsequently, linkage maps were generated for a bi-parental and a self-pollinated population with ‘Red New Dawn’ as the common maternal parent. The densest map, for the selfed parent, had 1929 SNP markers on 25 linkage groups, covering 1765.5 cM at an average marker distance of 0.9 cM. Synteny with the strawberry (*Fragaria vesca*) genome was extensive. Rose ICM1 corresponded to *F. vesca* pseudochromosome 7 (Fv7), ICM4 to Fv4, ICM5 to Fv3, ICM6 to Fv2 and ICM7 to Fv5. Rose ICM2 corresponded to parts of *F. vesca* pseudochromosomes 1 and 6, whereas ICM3 is syntenic to the remainder of Fv6.

## Introduction

Garden roses are tetraploid woody perennials from the genus *Rosa* (family *Rosaceae*, subfamily *Rosidae*). Roses have been cultivated since 5000 years, for ornamental purposes but also for food products (hips and petals), pharmaceuticals and perfumes.^1–3^ The high popularity of roses, the range of uses and intensive breeding activities have resulted in numerous cultivars. The frequent hybridization with and introgression from wild species into cultivated roses^4–6^ have led to a complex taxonomy.^7,8^ The result of these introgression events may be that some chromosomal regions may be more diverse than others among garden rose cultivars,^9,10^ and possibly genetically differentiated from those in cut rose.

Diploid rose species have 14 chromosomes (2*n*=2*x*=14), whereas tetraploids have 28 chromosomes (2*n*=4*x*=28). Depending on the similarity between particular chromosomes in a tetraploid rose cultivar, the mode of inheritance could be tetrasomic (random pairing of the four homologous chromosomes), disomic (strict preferential pairing of chromosomes, as in allopolyploids) or intermediate between these two,^11,12^ but this has not been quantified in rose. In the case of tetrasomic (random), pairing the chromosomes can form quadrivalents in meiosis, after which (parts of) sister chromatids may find themselves in the same gamete during the second meiotic division. This phenomenon is termed double reduction. It increases the homozygosity in gametes compared with what would be expected under random chromosome segregation.^13^ Double reduction is contingent on the occurrence of quadrivalents and on the occurrence of a crossover between the centromere and the observed locus;^14^ therefore, the frequency increases towards the distal ends of chromosomes.

A rose linkage map is useful for the study of rose genetics. A dense linkage map will enable localization and mapping of differentially expressed transcripts,^15^ genes and quantitative trait loci (QTLs) for important traits such as disease resistances^16,17^ and various ornamental traits,^1,4,18–20^ as a step towards marker-assisted breeding (for example, ref. 21). In tetraploids, multi-allelic markers, such as microsatellites, can amplify up to four different alleles in a single genotype, but single-nucleotide polymorphism (SNP) markers are biallelic, so we can only distinguish two alleles. Thus, a single microsatellite marker is generally more informative than a single SNP marker^22^ if it is scored co-dominantly.^23,24^ Development of highly polymorphic microsatellite markers^25–29^ and techniques for the determination of allele dosage of microsatellite markers^30–32^ have been developed, but it is still a laborious and time-consuming analysis. In contrast, thousands of SNP markers can be detected in parallel in one hybridization step, which compensates by far for the lower information content of a single SNP. In addition, scoring is largely automated as well. Recently, the 68k WagRhSNP array has been developed for roses,^33^ opening up the possibility to produce high-density linkage maps in rose.^34^

Linkage maps in the genus *Rosa* have been produced for several diploid populations^16,17,27,35–42^ and a few tetraploid populations.^12,26,42–44^ The average distance between markers in those linkage maps was large, except in the integrated consensus map,^40^ where it was 0.88 cM after combining information from five populations. For tetraploid maps the average marker distance was 2.4 cM^44^ to 5.3 cM.^43^ The maximum distance between adjacent markers in the tetraploid maps was 16 cM^44^ to 39 cM.^42^ It is unclear whether all homologous chromosomes and chromosomal regions were represented in the linkage maps.

Using SNP markers enables improving map coverage and density, and at the same time reduces the efforts and costs involved in producing the linkage map. Currently,TetraploidMap is the only publicly available software for mapping in autotetraploids.^45^ Although it can include markers that segregate as simplex×nulliplex as well as duplex×nulliplex markers, it is restricted in the number of markers and needs manual interaction and visual inspection, which limits its implementation.^46,47^ A new version has just been developed.^48^

The aim of this study was to generate the first high-density genetic linkage map for tetraploid garden rose, using an approach that starts with constructing separate homologs.^34^ SNPs were genotyped using the rose WagRhSNP array and the SNP dosage was estimated by fitTetra.^49^ We also developed a strategy to disentangle offspring from different crosses in the absence of some of the parents, using the large amount of SNP scores and the information therein. The results enabled a detailed analysis of synteny of the rose genetic linkage maps with the woodland strawberry genome sequence.

## Materials and methods

### Mapping population(s)

A set of 177 seedlings was obtained that was intended to be an F1 mapping population from a cross between the two garden rose cultivars ‘Red New Dawn’ (RND, mother) and ‘Morden Centennial’ (MC). However, as will be shown in the Results section, the seedlings proved to have different origins; two subpopulations will be referred to as RND×RND (selfing of RND, 103 individuals) and RND×UP (RND×UP, 74 individuals). Genomic DNA was extracted from freeze-dried young leaves of RND, MC and 177 seedlings with the Qiagen DNeasy Plant Mini kit (250) (Qiagen, Venlo, The Netherlands) following the protocol used by Esselink *et al*.^25^ The seedlings were grown on their own roots in a greenhouse in Wageningen, The Netherlands.

### Microsatellite markers

Microsatellite or simple sequence repeat markers were used to align the numbering of linkage groups to the Integrated Consensus rose linkage Map (ICM) map.^40^ Thirty-two markers (from Esselink *et al.*,^25^ Hibrand Saint Oyant *et al.*,^27^ Yan *et al.*^37^ and Meng *et al*.^50^) were chosen to genotype the offspring (Supplementary Table ESM1). Amplification was in 10 μL containing 1 μL of 8 ng μL^−1^ DNA, 5 μL multiplex kit (Qiagen) and 4 pmoL of forward (labeled) and reverse primers, using 15 min. denaturation at 95 °C. followed by 30 cycles of 94 °C for 30 s, ramp 1 °C s^−1^ to 50 °C, 50 °C for 30 s, ramp 1 °C s^−1^ to 72 °C, 72 °C for 120 s and a final extension at 72 °C for 10 min. One microliter of 100× diluted PCR product was mixed with Hi-Di formamide containing GeneScan-500 LIZ size standard (Applied Biosystems, Foster City, CA) and separated on an ABI 3730. The output was analysed using Genemapper 4.0 (Applied Biosystems). The allele dosage was scored quantitatively.^31^ Nevertheless, we did not include the microsatellite markers in the genetic linkage maps as due to missing data points their location would be imprecise compared with that of the SNPs.

### SNP markers

Generation of SNP data, dosage scoring and genotype calling, and map construction followed the steps described in Smulders *et al*.^34^ Step 1 concerned generating the SNP data; in step 2, these were inspected and filtered, genotypes were called, and their quality was checked; and in step 3, markers were assigned to homologs, linkage maps were constructed and checked for consistency. For genotyping, we used the WagRhSNP Axiom SNP array,^33^ which contains 68 893 SNPs probed from both directions. Hybridizations of all offspring plants and the parents were performed by Affymetrix (Santa Clara, CA, USA). In a tetraploid, a SNP with two alleles, a and b, can be present in five different dosages: aaaa (nulliplex for b), aaab (simplex), aabb (duplex), abbb (triplex) and bbbb (tetraplex). We used fitTetra^49^ to score allele dosages. FitTetra uses a mixture model approach. The two probes for each SNP were fitted as independent markers. Missing scores were assigned if the dosage of a sample could not be assigned with sufficient confidence (assignment probability <0.95) or if the total signal intensity was too low.

In step 2, we initially judged for each SNP if the quality was acceptable based on: (a) the number of missing data, (b) the number of conflicting scores for replicated samples, (c) match of F1 progeny segregation to 1 of the 20 expected disomic or tetrasomic segregation patterns and (d) match of parental dosages with the F1 segregation. We selected only those SNPs for which both probes passed this quality check, but only if <4% of the F1 dosages differed between the probes and the results of both probes matched the same segregation pattern. The dosage with the highest probability was selected.

The results of step 2c, the match for possible segregation patterns, prompted us to recheck whether all seedlings were really offspring of the presumed parents, as for a large number of SNPs the segregation in the progeny did not agree with an F1 and/or the expectation based on the scored parental dosages. A principle component analysis (PCO) plot of the 177 seedlings using pairwise genetic distances based on all markers, made with NTSYS 2.10,^51^ produced one cloud of samples. We then generated PCO plots of the seedlings based on pairwise genetic distances calculated only for SNPs that were monomorphic in one of the intended parents. Based on these plots, two underlying, admixed populations were manually separated. We then repeated step 2 for these two populations.

### Linkage map construction

We followed Bourke *et al*.^13^ in naming the segregation types of markers based on the dosages in both parents. For step 3, the linkage map construction, SNPs with the following segregation types are most informative: simplex×nulliplex (one allele in one parent, allele absent in the other, segregation 1:1), duplex×nulliplex (two copies of the allele in one parent, absent in the other) and simplex×simplex (one copy of the SNP allele in both parents). We first assigned simplex×nulliplex markers to linkage groups. As the estimates of recombination frequencies and log of odds (LOD) scores for simplex×nulliplex markers in coupling phase are the same for tetraploids as for diploids,^23^ JoinMap 4.1^52^ could be used for linkage group detection. The only exception is when double reduction occurs, but this is expected to occur only at a low frequency. For instance, Bourke *et al*.^13^ found frequencies ~6% or more towards the distal regions of the chromosomes in autotetraploid potato. Our simplex×nulliplex SNPs were analysed using JoinMap in coupling phase for each parent separately in the cross-pollinated population and selfed population. A *χ*^2^ goodness-of-fit test was performed on the segregation data of all markers, and the markers deviating significantly (*P*<0.05) from the expected 1:1 or 1:2:1 ratios were removed from the analysis. The SNPs were grouped into linkage groups on the basis of a logarithm of odds ratio (LOD for independence >4).

The SNPs in the linkage groups were separated into four homologous chromosomes using the assigned phase and the recombination frequency estimates, where possible. The recombination frequency between markers at the same position on different homologs is expected to be 1/3,^53^ which corresponds to ~39.5 cM when using the Kosambi mapping function. If markers from different homologs are mapped together (as if they were on the same homolog), gaps of ~40 cM are therefore expected between different homologous chromosomes; such gaps were identified and used to separate the homologs. Each parental map is expected to have a total of 28 linkage groups, corresponding to the seven chromosomes and four coupling phase linkage groups per chromosome. To connect homologs within parental genomes and chromosomes across parental genomes, we subsequently added so-called bridge markers to the simplex×nulliplex segregating markers: duplex×nulliplex markers, which segregate 1:4:1 in an autotetraploid, and simplex×simplex markers, which segregate 1:2:1. Recombination frequencies and LOD scores between these markers, as well as with the simplex×nulliplex markers, in coupling phase were obtained by maximum likelihood estimation from the pairwise frequencies of the dosages of the markers in the mapping population. These estimates were assembled in so-called ‘pairwise data files’ and imported into JoinMap. A grouping tree was generated on the basis of a LOD threshold of 4. For linkage map construction, we used the weighted least squares regression algorithm of Joinmap with default settings.

A second method to connect homologs was by determining which of the SNPs that mapped to different homologs were derived from the same contig. For this, we used the name of the SNP on the array (Supplementary Table 2 of Koning-Boucoiran *et al.*^33^), as it includes the contig number, followed by a number that indicates the position of the SNP on the contig.

Third, the homologs were also indirectly anchored by comparing the position of the contig DNA sequence from which the mapped SNP(s) were derived, with that of the most similar sequence in the *Fragaria vesca* genome^54^ version 2.0 (https://www.rosaceae.org/species/fragaria_vesca/genome_v2.0.a1). The BLASTN was done using a sliding window with wordlength *w*=9 and *w*=11, selecting the highest hit if above a cutoff E-value of 10^−5^. The more stringent *w*=11 BLASTN mapped only 34% of the markers that were mapped under the more relaxed *w*=9 settings, but the synteny pattern was clearer. This BLASTN analysis also enabled visualization of the synteny between rose and *F. vesca* using Circos.^55^

We adopted the linkage group numbering of the ICM.^40^ The assignment of linkage group numbers to our linkage maps was carried out using microsatellite markers from the ICM map, but the orientation of the maps generated here is that of the *F. vesca* pseudochromsomes. Linkage maps were drawn with MapChart.^56^

## Results

### A strategy to distinguish subpopulations based on selected SNP scores

In the 177 offspring plants of the intended cross RND×MC, only ~55% of the simplex×nulliplex SNP markers from RND fitted the expected segregation ratios, and only 17% of those from MC (not shown). Among these markers, we observed triplex and quadruplex allele dosages that were not expected, given the parental SNP dosages. Most signal intensities were within acceptable ranges, suggesting that it was not a technical issue but possibly due to the presence of outcrossed plants.

**Figure 1:**
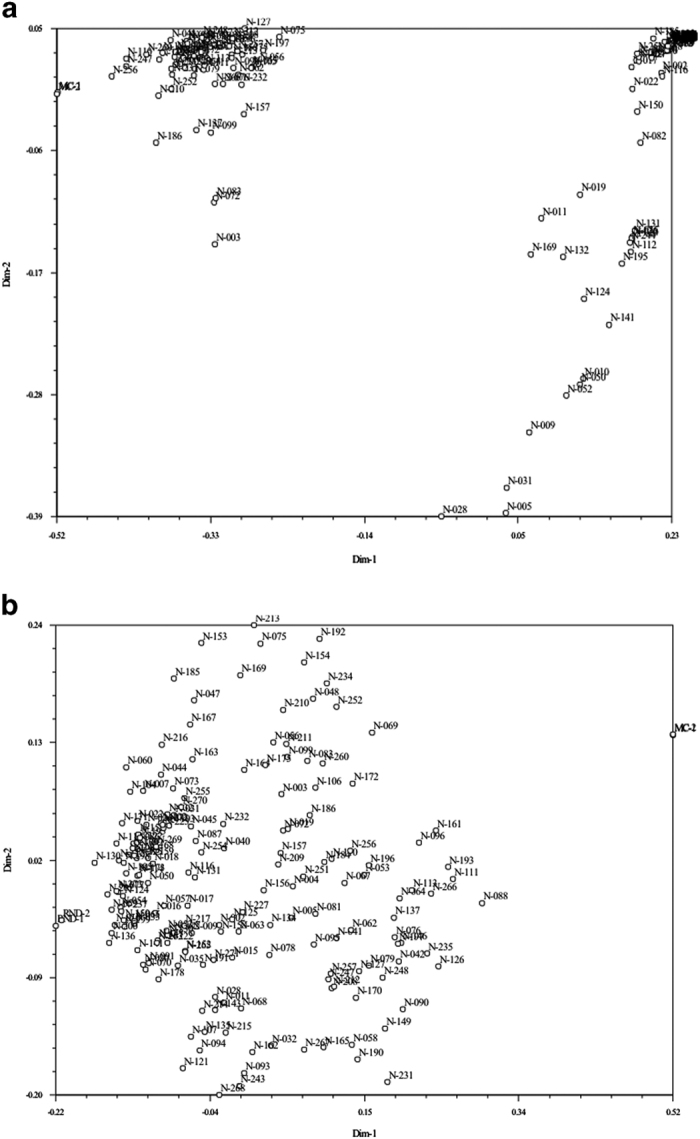
PCO analysis of the progeny based on pairwise genetic distances using only the SNP markers that were monomorphic (nulliplex or quadruplex) in RND (**a**, based on 18 683 markers) and MC (**b**, based on 17 935 markers). In (**a**), the first principal coordinate explained 52% and the second 29% of the variation; for (**b**), this was 56% and 27%, respectively. The results in (**a**) indicate that the population consists of two subpopulations, and two pollen donors have been involved in crosses: A (the cluster to the left, 103 offspring) and B (the cluster to the right, 74 offspring). The results in (**b**) give no indication that more than a single mother was involved.

We therefore went back to the SNP selection steps and included the SNPs that had been filtered out in step 2c based on expected segregation ratios. Visualizing the population structure with all markers in a PCO did not produce clear groups among the seedlings. To improve the resolution, we generated two PCO plots for all offspring using pairwise similarities based on selected SNP markers, namely, those for which RND (Figure 1a) or MC (Figure 1b) were monomorphic (nulliplex or quadruplex). These markers are uninformative for one parent and can show the genetic relationships due to the other parent(s) at a higher resolution. The PCO plots indicated that, as expected, there was no differentiation based on the markers that were informative from the maternal side (Figure 1b), but the paternally informative PCO (Figure 1a) plot indicated two clusters of samples, which was interpreted as two subpopulations. This points to two different fathers. Thus, we divided the initial population into two populations: population A (consisting of 103 offspring, the left cluster in Figure 1a) and B (consisting of 74 offspring, the cluster to the right in Figure 1a). In both populations, the genotype of MC could not explain the segregation in the progeny and thus it was rejected as the pollen parent. Population B was named RND×UP, a cross of RND with an ‘unknown parent’ (UP). On the basis of genotype configurations of RND and the offspring, the marker genotype for UP could be reconstructed.

For the larger of the two subpopulations (population A), the set of segregating markers that passed all quality criteria (except concordance with the—unknown—paternal parent) consisted of 13 941 markers, of which very few were simplex×nulliplex segregating markers. Not a single marker segregated duplex×nulliplex, but many segregated simplex×simplex. As the inferred dosage of the parents was the same for the vast majority of the markers, we assumed that this population may be the result of self-fertilization (selfing). To confirm this assumption, we looked at the single dosage markers in RND. If this population is derived from selfing, then all SNPs that segregate simplex×nulliplex from RND in the RND×UP population would have to segregate as simplex×simplex in this population. Of the 1411 simplex×nulliplex markers in RND×UP, 1099 were also scored in population A and 1061 of them (96.5%) segregated as simplex×simplex. Of the 943 simplex×simplex markers in the RND×UP, population 689 were also scored in population A and for all markers the segregation was consistent with simplex×simplex. Therefore, we concluded that population A most likely originated from selfing and named it RND×RND. As an additional indication that population RND×RND arose from selfing, the progeny of RND×RND had a significantly lower heterozygosity than RND (0.55 vs 0.69; *P*=0.001) across all markers.

### Linkage map construction

For the RND×UP population, we produced linkage maps for both parents. A total of 2513 SNPs (1411 simplex×nulliplex, 942 simplex×simplex and 160 duplex×nulliplex markers) were used for the construction of a genetic linkage map of RND, whereas for the UP map, 1760 SNPs (615 simplex×nulliplex, 942 simplex×simplex and 203 duplex×nulliplex markers) were used.

The resulting RND linkage map included 1120 SNPs assigned to 23 linkage groups with a median distance between markers of 0.96 cM and a maximum distance between two consecutive markers of 17.5 cM. The linkage groups varied in size from 12.5 to 94.4 cM (Table 1), adding up to a total map length of 1082.8 cM. The 23 linkage groups have been assigned to the seven chromosomes of the ICM linkage map. For two chromosomes, all four homologs were present; for five chromosomes (1, 3, 4, 6 and 7), one homolog was missing.

The resulting UP linkage map included 524 SNPs (Table 1) distributed over 18 linkage groups. The length varied from 9.1 to 107.6 cM with a mean interval distance between loci of 1.4 cM and a maximum distance between two consecutive markers of 13.2 cM. In total, this linkage map spanned 740.8 cM (Table 1). Only for chromosome 6, all four homologs were present; for chromosomes 2, 4, and 7, one homolog was missing; for chromosomes 3 and 5, two homologs were missing; and for chromosome 1, three homologs were missing.

**Figure 2:**
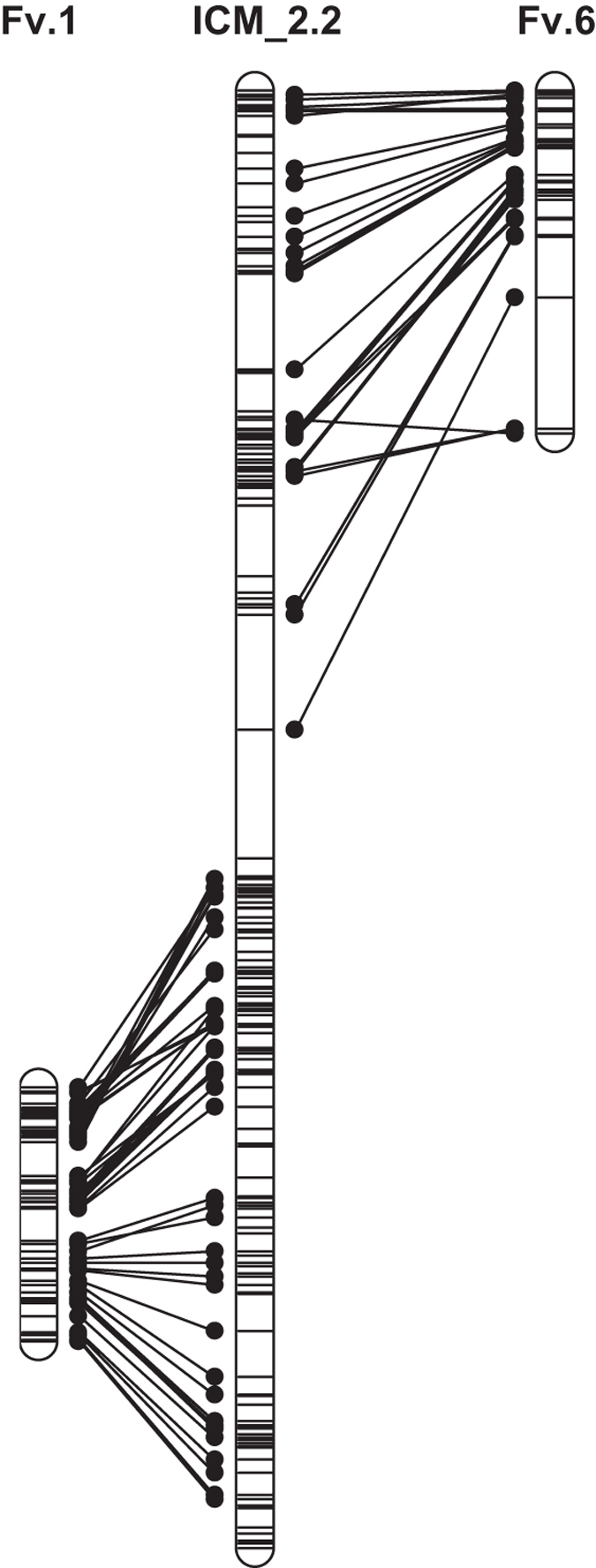
An example of the colinearity of the rose and strawberry genomes. Rose linkage group ICM2 homolog 2 (ICM_2.2) of the map of RND from the RND×RND population as constructed with SNP markers in the center, and the synteny with two *Fragaria vesca* pseudochromosomes (Fv6 and Fv1) indicated to the left and to the right. ICM_2.2 positions in cM; Fv in Mbp. Marker names and positions, and the MapChart files of the complete RND linkage map are in Supplementary zipfile ESM5, all BLASTN hits to *F. vesca* in Supplementary file ESM6. Using MapChart,^56^ maps of all other homologs can be visualized by opening the corresponding MapChart file.

Using the selfed progeny, a denser RND linkage map was produced (one homolog is shown in Figure 2). Here 1929 SNPs were mapped to 25 linkage groups, spanning 1765.5 cM (Table 1). The length of the linkage groups varied from 15.2 to 118.2 cM. The average marker density was 0.9 cM and the maximum distance between two consecutive markers was 25.4 cM. On the linkage map of this selfed population, most of the markers were simplex×simplex. Simplex×nulliplex markers did not occur, with the exception of six S×N markers that were mapped to the linkage group ICM3.

As expected, most (14%) markers were shared between the two RND maps (Table 1). The largest number of shared markers among maps was on ICM3, which was also the linkage group with the largest number of markers in all maps (24.2–26.1% of all mapped markers). No markers were shared for ICM1. The three parental maps have been aligned using the common markers, as far as the homologs shared markers. Only one homolog is shown here (ICM2.2 in Figure 2, see below) but all homologs can be drawn in MapChart^56^ using the linkage group files provided in Supplementary zipfile ESM5.

### Synteny with *Fragaria*

For the parental RND linkage map, contig sequences of 379 SNP markers were successfully blasted to the strawberry genome, under stringent conditions (Supplementary Table ESM3), whereas 1112 markers mapped under less stringent BLAST conditions (at *w*=9). For the UP map, 191 and 510 markers could thus be located on the *F. vesca* genome sequence (Supplementary Table ESM4). For the RND map of the RND×RND population, 651 and 1800 markers had a hit (Supplementary Table ESM2). Under stringent conditions, a total of 1221 markers from the three maps were located on the *F. vesca* genome sequence (Table 2).

Most of the markers from one rose linkage group (84–98%, except for ICM2) were located on a single *F. vesca* pseudochromosome, and in largely the same order (colinear), which indicates a high level of macro-synteny between rose and strawberry. This picture emerged both under the stringent BLAST conditions (wordlength 11, Table 2) and under less stringent conditions (wordlength 9), which gave a hit for 3422 markers, that is, almost three times as many, but with a low number of possibly accidental similaries on all other pseudochromosomes (Supplementary Tables ESM2–4).

**Figure 3:**
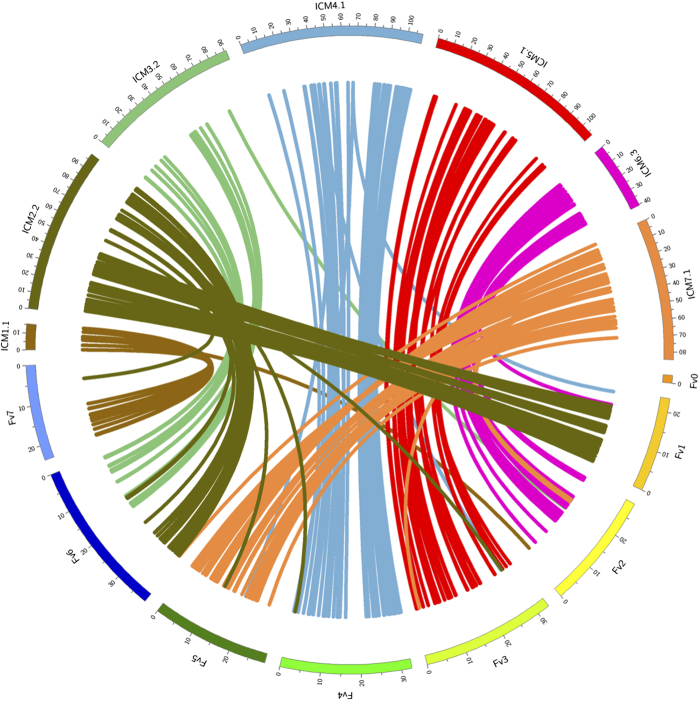
Circos plot of the synteny between rose and strawberry, on the basis of the contigs of the SNP markers of the RND×RND map that were BLASTed against the *Fragaria vesca* genome assembly (v2.0.a1), wordcount=11. The rose ICM homolog containing most mapped markers per linkage group are shown, with distances in cM. Fv numbering refers to *F. vesca* pseudochromosomes with distances in Mb. Marker names and positions, and the MapChart files of the complete RND linkage map are in Supplementary zipfile ESM5, all BLASTN hits to *F. vesca* in Supplementary file ESM6.

Garden rose ICM1 corresponded to *Fragaria* pseudochromosome 7 (Fv7), ICM4 to Fv4, ICM5 to Fv3, ICM6 to Fv2 and ICM7 to Fv5. Rose ICM2 corresponded to parts of *Fragaria* pseudochromosomes 1 and 6 (Figure 2), whereas these parts were largely colinear. Rose ICM3 is the remainder of Fv6 (Table 2, Supplementary file ESM6). Generally, the Circos plot (Figure 3) is consistent with the results of Gar *et al*.^44^ and Kirov *et al*.,^57^ but our dense map makes it possible to locate the breakpoint in Fv6 (Figure 2) between 18 and 20 Mbp. A small piece (from 17 to 21 Mbp) of Fv1 may be present at the end of ICM2. The resolution is not sufficient to detect with certainty whether other small translocations exist. The Circos plot also shows that the two genomes are largely colinear.

## Discussion

We developed a strategy to disentangle an admixed offspring from two crosses, even in the absence of one of the parents, using a large number of SNP dosage scores from the WagRhSNP array and the segregation information therein. We subsequently generated high-density genetic maps for two tetraploid garden rose populations, with a SNP marker density below 1 cM between markers, which up to now has only been obtained in the ICM map^40^ by integrating five different maps.

During construction of the linkage maps, we detected a large number of presumed F1 offspring plants that were not in agreement with the genotypes of the putative parents, beyond the small number of off-type offspring that is a common nuisance in breeding (if problems in population uniformity are reported at all). We used subsets of SNP markers for which no segregation was expected from one of the intended parents, as a strategy to detect genetic groups related to the other parent(s). With this relatively simple procedure, and with the information present in the vast number of SNP marker data available, we were able to reconstruct two subpopulations with different parentage even in the absence of genotype information of one putative parent.

One of the two populations was the result of a selfing of the mother, variety RND. This was concluded based on several lines of evidence as follows: the absence of simplex×nulliplex segregating markers, the fact that >96% of the RND markers that segregated as simplex×nulliplex in the RND×UP population behaved as simplex×simplex in this offspring, and the 20% lower average heterozygosity compared with the mother. To date, no studies on self-compatibility in garden roses have been published, whereas breeders’ experiences are confidential. Self-pollinated flowers of the diploid *R. rugosa* Thunb. wilt after pollination, suggesting the existence of gametophytic self-incompatibility,^58^ but Nybom *et al*.^59^ indicated that polyploid species appear to be fully self-fertile. Self-fertility has been overlooked in breeding, and our finding indicates a need to better manage pollinations while making controlled crosses in a greenhouse. On the other hand, self-fertility makes it possible to fix highly valued traits by one or two rounds of selfing. Up to now, it is unclear to what extent selfing has played a role in commercial breeding in roses.

We constructed three linkage maps for garden roses employing simplex×nulliplex, simplex×simplex and duplex×nulliplex segregating SNP markers. The largest linkage map (of RND from the selfed RND population) included 1929 loci and covered 1765.5 cM with an average marker distance of 0.9 cM. Compared with earlier tetraploid rose maps with average marker distances from 2.4 cM (homologs integrated^44^) to 5.3 cM (map per homolog^43^), homolog coverage and marker density are much improved. Some homologs are still missing, and this may not be random, as, for example, only 15 cM of ICM1 was found in the densest RND map, whereas all other linkage groups were covered by four homologs (albeit not necessarily all complete). It may be that the level of polymorphism varies among chromosomes, possibly related to the origin of each of them in tetraploid rose, which may be composed of chromosomal segments from up to 10 species.^3,10^ On the other hand, it cannot be ruled out that breeding and selection (with, for example, one or two rounds of unforeseen selfing) have led to regions of (relatively high) homozygosity, as was observed in octaploid strawberry.^60^

Larger mapping populations will greatly increase the statistical power for QTL analyses. Software specifically being developed for polyploid maps,^48^^,^^61^ will enable using all markers with other segregation types, which have now remained unused, and integrating homologs. The ability to reliably generate separate haplotypes of separate homologs will contribute to our understanding of polyploid inheritance, to the estimation of heterozygosity and to determining the population structure of polyploids.^62^

The genus *Rosa* is closely related to the genera *Fragaria*^63^ and *Rubus*^64^ in the subfamily Rosoideae, and thus the *F. vesca* genome assembly can be used as a reference for the validation of genetic linkage maps in rose,^44^^.^^65,66^ Our synteny analysis indicated a high level of conservation between the rose and strawberry genomes. The majority of the markers that mapped on one linkage group in rose had their highest sequence similarity with the sequence of a single pseudochromosome of strawberry, with the exception of rose linkage groups 2 and 3: their markers corresponded to both strawberry pseudochromosomes 1 and 6, indicating that translocations have occurred. There is also a high degree of colinearity (as visualized in Figures 2 and 3). The macro-synteny observed in this study is in agreement with the study of Gar *et al*.^44^ in rose. Other studies between members of the *Rosaceae* also indicated a high level of synteny and detected strongly conserved syntenic regions among the genera *Malus, Fragaria* and *Prunus.*^67,68^ The high level of synteny can be used to obtain positions of non-segregating markers or contigs and to find candidate genes both in QTL regions and around significantly associated markers in genome-wide association studies (Schultz *et al.*, submitted). In addition, it offers an additional means of linking markers between segregating populations or genome-wide association studies, as next to multiple SNPs on one contig (up to eight SNPs on the array were derived from a single rose contig^33^) markers may also be linked between physically neighboring contigs using the Fragaria genome sequence.

The WagRhSNP array is already being used to generate a diploid rose map or *R. chinensis* ‘Old Blush’×*R. wichurana* with greatly increased marker density.^69^ Very dense maps of markers based on the genetic segregation data will be very helpful in the assembly of the scaffolds of the rose genome^70^ based on bioinformatics into correct pseudochromosomes, as was the case in the revision of the apple genome.^71^

### Conclusions

We developed a strategy for distinguishing subpopulations that share one parent based on SNP segregation data that are monomorphic for one of the presumed parents. Using this strategy, we confirmed that selfing occurs in garden rose, which may open new possibilities for rose breeding. We used the SNP array data to produce three dense genetic linkage maps for garden roses, which in comparison with previous rose maps have significantly improved coverage of homologs and increased marker density. With these maps, we showed that synteny of rose and strawberry is extensive. The colinearity of these genomes will be very useful in finding candidate genes underlying QTLs and regions identified using genome-wide association studies.

## Figures and Tables

**Table 1:** Linkage map length and number of markers for the paternal RND and maternal UP maps of the RND×UP population and the map of RND from the selfed population

*Linkage group*	*Homolog*	*UP map (from RND×UP cross)*		*RND map (from RND×UP cross)*		*RND map (from RND selfed population)*	
		*Map length (cM)*	*Number of markers*	*Map length (cM)*	*Number of markers*	*Map length (cM)*	*Number of markers*
ICM1	H1	35.9	45	12.5	17	15.3	44
	H2			23.3	9		
	H3			28.2	7		
	H4						
ICM2	H1	107.2	63	30.7	25	115.3	102
	H2	66.3	66	58.6	68	98.7	261
	H3	9.1	17	76.3	86	64.8	99
	H4			56.1	102	34.4	43
ICM3	H1	73.3	64	58.6	43	67.6	46
	H2	15.8	18	72.3	83	91.2	72
	H3			36.8	30	82.7	63
	H4					79.7	50
ICM4	H1	86.9	70	49.4	67	108.7	127
	H2	15.5	8	33.2	49	43.4	76
	H3	12.5	6	20.8	34	34.7	19
	H4					18.3	55
ICM5	H1	71.8	39	67.4	50	107.6	137
	H2	27.1	31	52.1	54	91.5	65
	H3			23.2	28	87.7	54
	H4			19.0	16	118.2	46
ICM6	H1	21.6	7	94.4	77	79.2	92
	H2	16.0	11	85.1	73	78.0	49
	H3	43.4	16	58.5	68	40.7	102
	H4	43.0	17			45.8	52
ICM7	H1	32.3	14	68.2	47	84.5	141
	H2	42.5	20	23.4	34	31.9	28
	H3	20.6	12	34.7	53	71.5	66
	H4					74.1	40
Total		740.8	524	1082.8	1120	1765.5	1929

Abbreviations: RND, Red New Dawn; UP, unknown parent.

Homologs have the same number as far as they shared markers.

**Table 2:** Synteny with strawberry

*Rose linkage group (ICM)*	*Fragaria vesca pseudochromosome*						
	*Fv1*	*Fv2*	*Fv3*	*Fv4*	*Fv5*	*Fv6*	*Fv7*
1	1	2	0	1	0	0	**47**
2	**160**	0	1	2	10	**155**	4
3	18	3	1	1	3	**132**	0
4	1	0	1	**173**	2	1	0
5	0	0	**166**	2	6	2	0
6	1	**195**	3	0	0	0	0
7	6	2	2	2	**114**	1	0

Number of markers of the three garden rose SNP linkage maps that could be placed on the *Fragaria vesca* pseudochromosomes. For this, the rose sequence contigs from which the SNPs had been derived were BLASTed to the *F. vesca* genome sequence v2.0 with wordlength 11, and the best hit was used if above the cutoff E-value of 10^−5^, otherwise they were considered not mapped. In bold are the groups of markers that represent the most likely syntenous linkage groups.

## Notes

Supplementary Table ESM1

Supplementary Table ESM2-4

Supplementary zipfile ESM5

Supplementary file ESM6
